# The Role of Europe’s Schools of Public Health in Times of War: ASPHER Statement on the War Against Ukraine

**DOI:** 10.3389/phrs.2022.1604880

**Published:** 2022-03-16

**Authors:** Lisa Wandschneider, Yudit Namer, Nadav Davidovitch, Dorit Nitzan, Robert Otok, Lore Leighton, Carlo Signorelli, John Middleton, Jose M. Martin-Moreno, Laurent Chambaud, Henrique Lopes, Oliver Razum

**Affiliations:** ^1^ Department of Epidemiology and International Public Health, School of Public Health, Bielefeld University, Bielefeld, Germany; ^2^ School of Public Health, Ben-Gurion University of the Negev, Be’er Sheva, Israel; ^3^ The Association of Schools of Public Health in the European Region (ASPHER), Brussels, Belgium; ^4^ ASPHER Honours’ Committee, Brussels, Belgium; ^5^ Department of Preventive Medicine and Public Health, Medical School and INCLIVA, University of Valencia, Valencia, Spain; ^6^ EHESP School of Public Health, Rennes, France; ^7^ Public Health Unit, Institute of Health Sciences, Catholic University of Portugal, Lisbon, Portugal

**Keywords:** public health, war, ASPHER, Ukraine, healthy and peaceful societies

War has devastating impacts on the health of populations. The consequences of war are multidimensional, affecting social life and health infrastructure, as well as environmental health. Along with immediate and long-term effects on the physical and mental health of all those involved, the consequences of war have the greatest impacts on vulnerable and marginalised groups [1–3]. In addition, war and armed conflicts forcibly displace people, creating additional public health problems throughout the displacement, flight and/or migration trajectory. The Association of Schools of Public Health in the European Region (ASPHER) reminds the attacking military of their obligation to the United Nations’ Humanitarian Law and WHO Resolution on Attacks on Health Care [4] that aims at ensuring that essential life-saving health services, including public health, are provided to emergency-affected populations unhindered by any form of violence or obstruction.

Public Health defaults to a pro-peace approach as ASPHER has outlined before in position papers relating to events such as the rights abuses in Turkey towards medical professionals following their stand against a military operation in Afrin [5, 6], the Israel-Palestine conflict [7], and the role of Schools of Public Health (SPHs) in peacebuilding [8]. Accordingly, ASPHER strongly condemns the military action against Ukraine that has claimed human lives and destroyed civilian infrastructure. We stand in solidarity with the Ukrainian people as well as with dissenting Russian citizens who object to this military aggression. ASPHER expresses its deepest concern about the impact of the war on the health and wellbeing of the Ukrainian people and other potential victims, as well as European and international society.

Schools of Public Health are entities capable of supporting civil society with critical skills and competences during conflict. In addition, SPHs are a major partner to national and international health organisations. ASPHER, as a network representing SPHs and public health training institutions in Europe for over 50 years, takes a stand as a credible source of expertise, solidarity, support and allyship for SPHs and the public health workforce of all affected countries. Guided by the principles for peacebuilding [8], ASPHER takes the following five-fold action:

(1) Discovery and dissemination of facts: ASPHER is creating an easily accessible mapping of knowledge, competences and skills that are of importance for SPHs and the public health workforce under conditions of conflict and war. Among them are emergency response and operations including environmental and infrastructure-related issues; public health surveillance and reporting systems in conflict zones; refugee and migrant health; the management of the Covid-19 pandemic [9]; other communicable diseases that emerge due to destruction of infrastructure; coordinated efforts with military personnel for public health interests; review protocols and competencies on radiation emergencies, nuclear explosion or nuclear leaks and how to protect population health in such eventualities. To support the effective implementation of these competencies and skills, ASPHER strengthens the existing cooperation in the SPH network, namely through sharing in scientific cooperation programmes, exchanges of scholars, distance learning and other forms of emergency digital training, assistance and scientific advice to health authorities, policymakers and other national or European or international entities, such as the United Nations Health Cluster in Ukraine.
(2) Diplomacy, mediation, and conflict transformation: ASPHER has started and will continue to mobilise and connect SPHs and public health colleagues from war-affected and neighbouring areas to help maintain dialogue between SPHs for an effective and appropriate response to public health challenges. In addition, ASPHER will give space to voices from inside Ukraine and people most affected by the military action to share their experiences through the Association’s communication channels, e.g., monthly newsletters and social media platforms.
(3) Solidarity and support: Specific attention will be paid to mobilisation of practical support networks for Ukrainians and residents of Ukraine. Firstly, this includes hosting scholars and students at risk in ASPHER member schools. ASPHER takes an active role in connecting affected scholars, facilitating exchange with SPHs that have experience in hosting researchers at risk and providing resources on potential partnering organisations (e.g., Scholars At Risk, Scholar Rescue Fund, CARA in the United Kingdom, Philipp Schwartz Initiative in Germany, PAUSE in France). Secondly, ASPHER will mobilise its substantial expertise in refugee health to support states that are likely to receive large numbers of refugees, potentially for an extended period of time. Such efforts will also include standing in solidarity with vocal peace activists in Russia.
(4) Use of health-related superordinate goals: Active monitoring and documenting human rights violations with probable consequence of ill health and stunting.
(5) Dissent and non-cooperation: ASPHER reiterates its position that it will stand against any activities undermining peacebuilding and will not cooperate with institutions that are associated with rights violations.

To effectively coordinate these actions, ASPHER has decided to create a task force^1^ monitoring public health impacts in Ukraine aiming to provide a solid foundation for prevention, preparedness and response to armed conflict.

Taking these actions is a natural continuation of ASPHER’s mission to promote “healthy and peaceful societies” based on the values of social accountability and health equity. Framing the situation within public health thinking broadens attention beyond immediate medical and humanitarian aspects of the conflict. Though extremely important, they are not enough. Social, economic and environmental determinants of health must be addressed to form a One Health sustainable response, strengthening local capacities with sensitivity to local communities’ needs. ASPHER calls on our members, partners, allies and networks to support these actions and join our efforts with the ultimate goal of an effective public health response to the war against Ukraine.

## Editorial Note

ASPHER is responsible and liable for the content. The statement was approved by an Editor-in-Chief but not externally peer reviewed.

The Global Network for Academic Public Health (GNAPH) endoreses the ASPHER statement. The Global Network is an alliance of the regional associations that represent schools and programs of public health around the world, including ASPHER.

Translated versions will be made available from: https://www.aspher.org.

## Footnotes

1 The task force is open to all ASPHER member schools. Please contact ASPHER secretariat at office@aspher.org if representative(s) of your institution wish to contribute.
